# Contactless heart rate measurement in newborn infants using a multimodal 3D camera system

**DOI:** 10.3389/fped.2022.897961

**Published:** 2022-08-09

**Authors:** Libor Svoboda, Jan Sperrhake, Maria Nisser, Chen Zhang, Gunter Notni, Hans Proquitté

**Affiliations:** ^1^Department of Pediatric and Adolescent Medicine, University Hospital Jena, Jena, Germany; ^2^Abbe Center of Photonics, Institute of Applied Physics, Friedrich Schiller University Jena, Jena, Germany; ^3^Group for Quality Assurance and Industrial Image Processing, Ilmenau University of Technology, Ilmenau, Germany; ^4^Fraunhofer Institute for Applied Optics and Precision Engineering IOF, Jena, Germany

**Keywords:** contactless monitoring, camera-based photoplethysmography, heart rate, newborn, 3D, vital parameters, neonatology

## Abstract

Newborns and preterm infants require accurate and continuous monitoring of their vital parameters. Contact-based methods of monitoring have several disadvantages, thus, contactless systems have increasingly attracted the neonatal communities' attention. Camera-based photoplethysmography is an emerging method of contactless heart rate monitoring. We conducted a pilot study in 42 healthy newborn and near-term preterm infants for assessing the feasibility and accuracy of a multimodal 3D camera system on heart rates (HR) in beats per min (bpm) compared to conventional pulse oximetry. Simultaneously, we compared the accuracy of 2D and 3D vision on HR measurements. The mean difference in HR between pulse oximetry and 2D-technique added up to + 3.0 bpm [CI−3.7 – 9.7; *p* = 0.359, limits of agreement (LOA) ± 36.6]. In contrast, 3D-technique represented a mean difference in HR of + 8.6 bpm (CI 2.0–14.9; *p* = 0.010, LOA ± 44.7) compared to pulse oximetry HR. Both, intra- and interindividual variance of patient characteristics could be eliminated as a source for the results and the measuring accuracy achieved. Additionally, we proved the feasibility of this emerging method. Camera-based photoplethysmography seems to be a promising approach for HR measurement of newborns with adequate precision; however, further research is warranted.

## Introduction

The vast majority of the preterm infants or compromised newborns require cardiorespiratory monitoring postnatally. Thus, compromised newborns or preterm infants have to be connected to transducers in order to provide continuous and accurate vital parameter monitoring. Well-established methods for monitoring vital signs such as heart rate (HR) and saturation of oxygen in the blood (SpO_2_) are electrocardiography (ECG) as well as pulse oximetry based photoplethysmography (PPG) (1, 2). To date both techniques are non-invasive, however contact-based, thereby requiring both adhesive sensors on the limbs and adhesive ECG electrodes on the chest wall. Attaching and re-attaching both the sensors and the electrodes is time-consuming. Furthermore, the sensors can lead to discomfort, stress, pain, epidermal stripping or even pressure ulcers especially in very low birth weight infants (VLBW) or extremely low birth weight infants (ELBW) due to their fragile skin (3–8). Furthermore, the wires' obtrusiveness may negatively impact the parent-child bonding process, particularly during kangaroo care (6). Additionally this kind of “traditional” monitoring has intrinsic limitations, namely inaccuracies due to frequent body motion artifacts (9) and lack of information, or even information loss due to sensor detachment or bending and twisting of the lead wires (10, 11). Furthermore, clinical conditions such as hypovolaemia or hypothermia can lead to vasoconstriction which can cause impairment of the blood flow to the limbs, and this will return measurement errors (12). There are also several further issues that these standard sensor systems/monitoring devices fail to address. Studies in perinatal settings have clearly shown that the time from birth to the measurement of an accurate HR using ECG or pulse oximetry often exceeds one or even two min (13–15). Very high rate of false alarms (87–93 %) stresses and desensitizes the caregivers and causes discomfort such as sleep deprivation for the neonates (16, 17).

We investigated a camera-based contactless method for monitoring the vital parameters of newborns or preterm infants. The camera-based sensor uses a novel contactless method of camera-based photoplethysmography (cbPPG). The method of cbPPG derives HR by using the same principles as contact PPG, detecting and amplifying subtle repetitive changes of the skin color occurring with each heartbeat.

In several studies, cbPPG was used under clinical settings to obtain the HR of newborn infants. Altogether a good correlation between cbPPG with ECG or pulse oximetry could be shown (4, 5, 7, 18–22). However, the acquired signal of the cbPPG is highly susceptible to three main confounders: motion artifacts, variable ambient light, and skin pigmentation (23, 24). Motion artifacts, however, can be a serious issue in contact-based systems as well (20).

The objective of this pilot study was to evaluate the feasibility and accuracy of the camera-based multimodal sensor in the clinical setting compared to the standard setting of a common pulse oximetry. Our approach is different to previous studies because it uses a combination of near-infrared (NIR) 3D imaging, 2D NIR and visible light (VIS) imaging. While 2D imaging is used to acquire multispectral video data, 3D imaging is employed to calibrate all relative camera positions in a common 3D coordinate system. This is used to track and evaluate the state of motion of observed subjects tested. While the measurement scheme itself is comparatively unique, its application in parallel to ongoing clinical operations was of noteworthy aspect. The entire study was performed with standard procedures without any significant change to daily clinical routines. This was achieved by constructing the sensor comprising the camera system in a highly adaptive rapid prototyping scheme (see section *technical details)*. From these primary tests, system-immanent and application-specific requirements can be derived. The aim was to check the proof of concept contactless 3D method, which was developed using prototyping processes. In addition to technological aspects we investigated influences of factors and characteristics such as motion artifacts, gestational age, sex, weight, skin temperature, or transcutaneous bilirubin on the accuracy of the measurements. Operational advantages and disadvantages during clinical practice are a matter of continued application and were only addressed heuristically in this study.

## Materials and methods

### Description of the multimodal 3D imaging system

**Figure 1:**
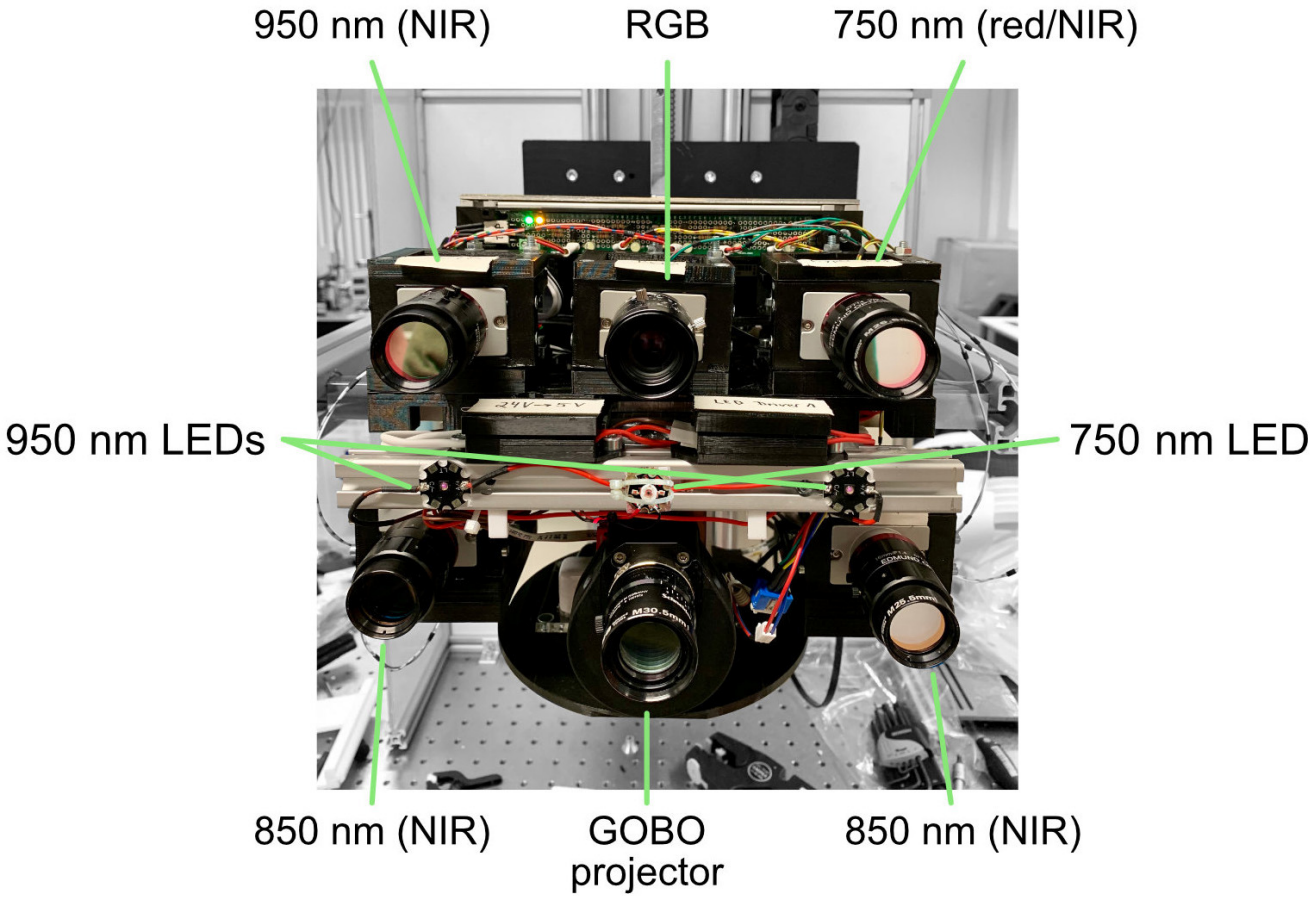
Interior of the multimodal 3D camera system with annotations for key components described in the main body of the text.

The multimodal 3D Sensor (Figure 1) is composed of a near-infrared (NIR) active stereo-vision camera setup based on GOBO (GOes Before Optics) projection technology (25, 26) for real-time 3D imaging, a color camera, and two NIR cameras at 750 nm and 950 nm for heart rate estimation. These NIR cameras are equipped with narrow-band band-pass optical filters with aforementioned central wavelengths and a full width at half maximum (FWHM) of 25 nm. The GOBO projector and the stereo-vision camera setup contain band-pass optical filters at 850 nm with a FWHM of 50 nm, so there is no crosstalk between projected GOBO patterns and the NIR cameras at 750 nm and 950 nm. Using a GOBO pattern set with a length of *N* = 10, a 3D image can be reconstructed using the correspondence search method of Dietrich (27). The 2D frame rate of the cameras of the stereo-vision setup was set to 300 Hz, so the stereo-vision setup can deliver 3D image data at a frame rate of 30 Hz. The color and NIR cameras are synchronized with the 3D image data stream with the same frame rate, and a light emitting diode (LED) array consisting of one LED at 750 nm and two LEDs at 950 nm is used for the homogeneous illumination in the NIR spectral range.

A geometric calibration of the cameras in the multimodal 3D Sensor was performed using the calibration approach (28) in order to estimate the geometric parameters of each camera. Based on the obtained camera parameters, an alignment of 3D image data with 2D color/NIR images can be realized by calculating the projection of reconstructed 3D data onto 2D image planes.

**Figure 2:**
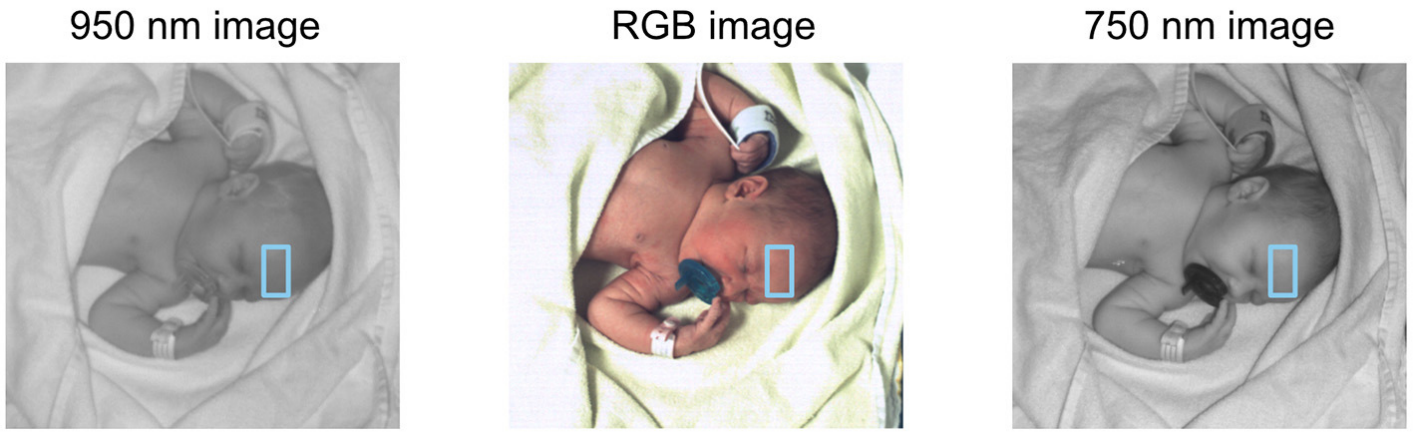
Image of a newborn in different spectral ranges. From left to right: 950 nm (NIR), RGB (visible light, true color), and 750 nm (edge of visible light to NIR). The ROI is defined by keypoints in the RGB image and transferred unto the two others.

For the estimation of heart rate from video data the approach in (29) was implemented, as explained in the following. In the first video frame, the face region of the newborn is detected in the 2D color image. In the face region of the color image a set of 2D keypoints (30) are detected, and a region of interest (ROI) is chosen on the newborn's forehead for the skin measurement in the NIR range (Figure 2). Since the 2D and 3D image data are aligned, the detected 2D keypoints and ROI can be directly located in the 3D space. From the second video frame, the 2D keypoints are tracked using the Lucas-Kanade method (31) in each color image. The 3D face pose of the newborn is then estimated from the 3D locations of the 2D keypoints tracked in the current frame and the 3D locations of these keypoints in the first frame. So, the 3D ROI created in the first frame can be transformed into the 3D coordinate system of the current frame using the estimated 3D face pose, ensuring that the ROIs located in different video frames refer to the same skin area. Furthermore, the 3D face poses estimated at different points in time can be used for motion analysis of newborns. The transformed 3D ROI then is located in 2D NIR images at 750 nm and 950 nm. Within the forehead ROI, the skin reflectance variations at both these wavelengths are measured for heart rate estimation.

In the NIR skin reflectance measurement the Eulerian video magnification (EVM) algorithm (32) is performed at all ROI pixels to amplify the temporal NIR intensity variations caused by heartbeats. Taking the possible heart rate range of newborns into consideration, the lower and upper limit of the digital band-pass filter in the EVM method are set to 1.5 Hz and 4 Hz, relating to heart rate values of 90 beats per min (bpm) to 240 bpm. From enhanced NIR video data at 750 nm and 950 nm two PPG signals are extracted by averaging the amplified temporal NIR gray value variations at all ROI pixels. Thereafter, a PPG signal fusion is realized by performing principal component analysis of both these PPG signals, thereby taking the first component as the final PPG signal (33). The power spectral density of this final PPG signal then is calculated, and the highest peak in the power spectrum is identified as estimated heart rate.

### Construction of the sensor device

It was essential for this study to operate the multimodal 3D imaging system without disturbing clinical procedures, being as non-obtrusive as possible for present staff, parents, and patients. Therefore, we employed rapid prototyping techniques in order to construct the camera system as a securely wall-mounted device with a 3D printed spherical casing. As a central skeleton structure we assembled a frame made of light-weight aluminum profiles (20 x 20 mm) and 5 mm thin aluminum sheets. This frame carried the three 2D cameras in an upper row and the stereoscopic 3D cameras as well as the GOBO projector in a lower row. Furthermore, internal electronics such as Arduino boards and a Raspberry Pi computer were carried in the back of the device. All illuminating LEDs were attached at the front on a central aluminum beam. To keep the overall weight of the sensor low while reducing fabrication costs we employed 3D printing for most other parts using polyactic acid (PLA) filaments. We printed stages or frames holding each camera, which were then screwed to the aluminum frame. To ensure proper cooling, we added four fans to the back of the device, similarly held by 3D printed frames. Finally, we 3D printed a spherical casing surrounding the entire sensor except for the front, where both cameras and LEDs were facing. To protect the front while allowing the camera to have optimal visibility, we used a laser-cut, plexiglass cover.

For secure wall-mounting we designed an exterior aluminum frame that could hook into standard issue clinical wall rails. In addition, the aluminum frame inside the device exhibited a vertical top beam extruding from the casing with a drilled eyelet. To protect the device in case it falls, the drilled eyelet can be attached to a steel wire, which is then hooked and tied into the wall rails. For ease of use, the sensor device could be controlled *via* an iPad browser application that sends commands to a wireless receiver of the sensor's Raspberry Pi computer. All acquired image data was transferred safely to a standalone PC *via* ethernet, directly from the cameras of the sensor.

### Setting

The study was designed as a single-center observational pilot study and proof of concept, including evaluation of the camera-based sensor in the clinical setting. The study was approved by the local Ethics Committee of the Jena University Hospital (Nr. 2020-1891-MV) and was conducted according to good clinical practice (GCP) and the Declaration of Helsinki.

**Figure 3:**
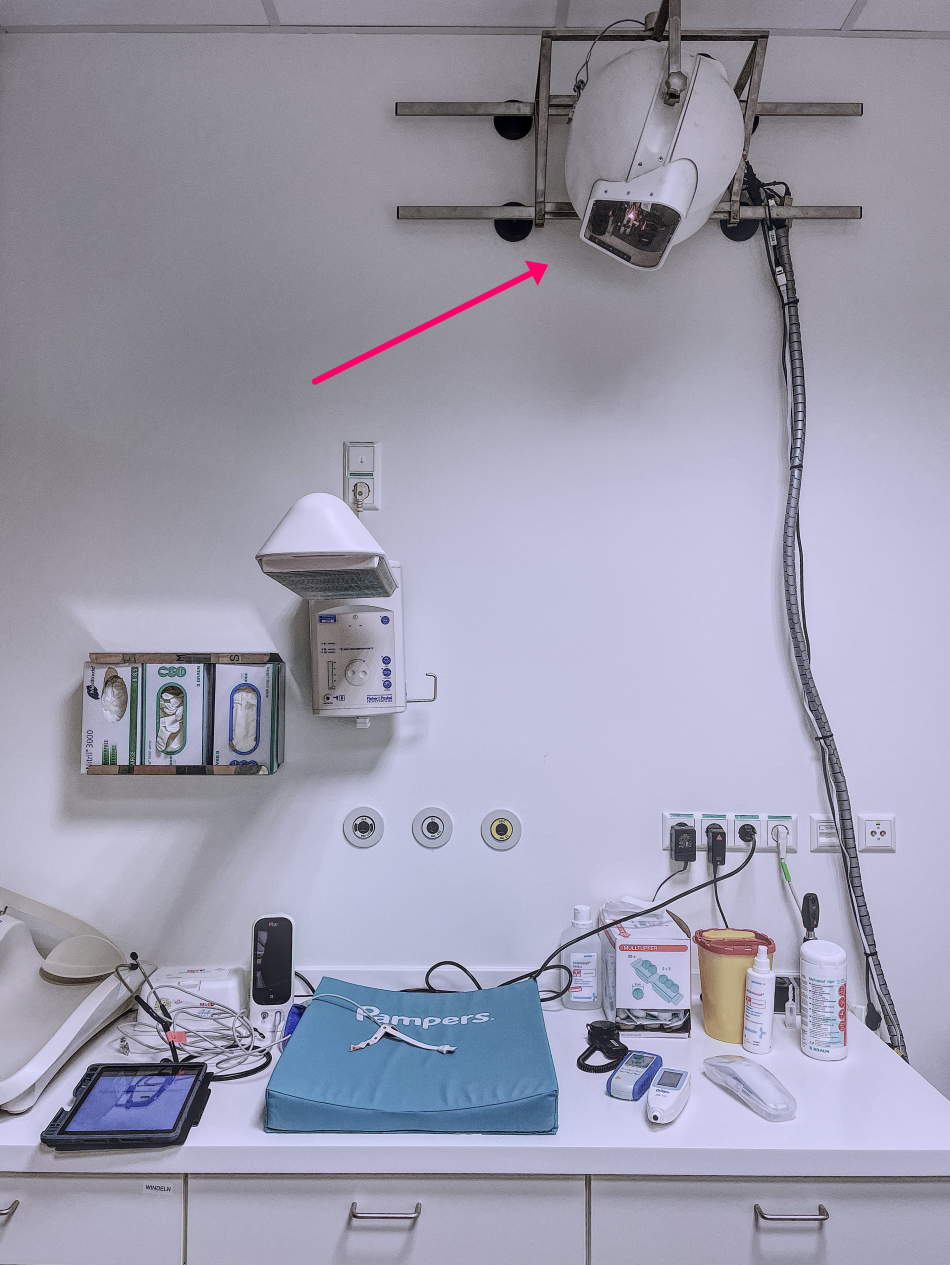
Camera-based sensor at the maternity ward, fixed to standard issue clinical wall rails.

The sensor was attached to the wall in the newborn examination room at a distance of 1.5 meters from the newborn examination table (Figure 3). This ensured operational safety as well as a field of view wide enough for the newborn's upper body to be visible in the multimodal 3D video feed. The regular hospital workflow was not disturbed.

After obtaining written informed consent from a legal guardian, the patient was brought to the examination room and placed on the newborn examination table. The pulse oximeter sensor was placed on the right hand (preductal) to avoid potential impact of a persistent ductus arteriosus (PDA). The ROI for cbPPG was the newborn's forehead so that there was no need to undress the patient. Most of the newborns were sleeping, but agitation and consequent motion could not be completely avoided. Multiple parallel measurements using the camera-based sensor and pulse oximetry-based monitoring system (*Masimo Rad-97 Pulse CO-Oximeter*^®^*, mcu: 1,068, processor: V 1.4.6.2 i-ss; tech board: 7e94)* as the reference standard were conducted during each visit. The averaging rate was 2-4 s. Data extracted from the Masimo device was saved in consecutive 2 s intervals with according time stamps. For comparability we used the same averaging rate of 2 s for the evaluation of the data obtained by the camera device. Acquired data from the camera-based sensor were continuously transferred to a PC. Prior to the first measurement, we conducted the exact time synchronization of the camera-based sensor with the Masimo pulse oximeter. This allowed synchronous reading and analysis of the data measured.

**Figure 4:**
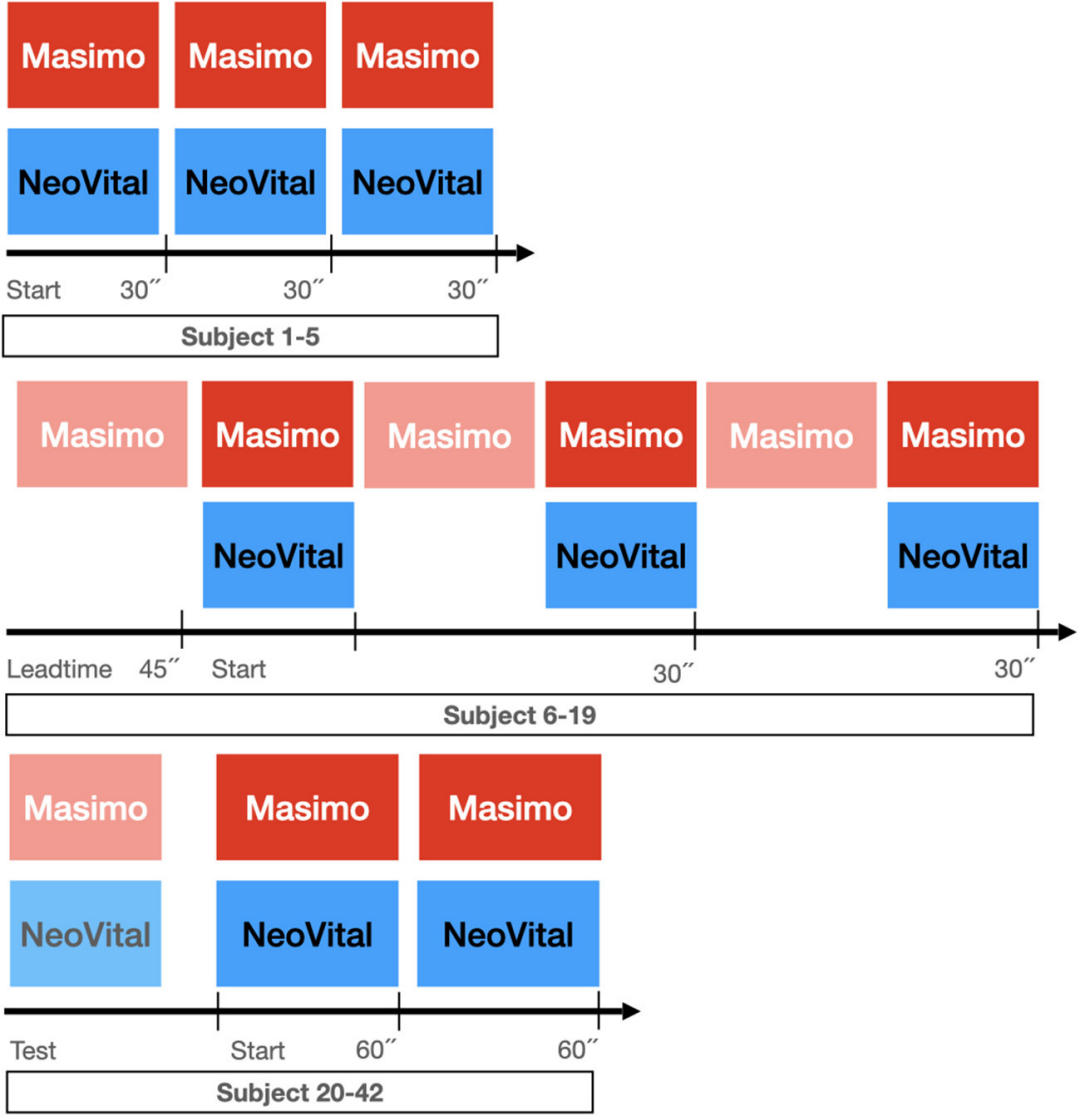
Adapted measurement time scale based on procedural evaluated application experience.

The measurements were carried out from 18th of January until 19th of March 2021 at the maternity ward of the University Hospital in Jena, Germany. Two measurement phases were carried out. In the first phase of our study, 19 newborn infants were examined. Initially, the recording period was set to 30 sec and sequentially repeated three times, starting both modalities simultaneously. After the first five subjects, we decided to adapt the study design because of many missing intervals in the first sec using the pulse oximeter. Thus, the pulse oximetry was connected 45 s prior to our camera-based sensor to both increase the amount and reliability of available data, sufficient for statistical analysis. In the second phase of the study, 23 newborn infants were examined. We sequentially obtained vital parameters twice for 60 sec, starting both measuring modalities simultaneously after running a short test of the signal of the pulse oximeter (Figure 4).

During the measurement, the values originated by the camera-based sensor were not visible to the investigator. The HR from the camera-based sensor was derived using a *post hoc* analysis, in order to compare the corresponding results afterwards. There were interruptions in the pulse oximetry measurement using the Masimo-sensor due motion artifacts that were detected later when the video was reviewed. Similarly, the video-based pulsatile signal was motion sensitive, too. Therefore, some intervals could not be analyzed because no data was produced. Hence, precondition for direct comparison of cbPPG-derived and PPG-derived values was the simultaneous recording of corresponding data at the same time. In line with the predefined protocol due to a large amount of both data and time required, a preliminary analysis was necessary. We found that in the first phase the calibration of the 3D camera system was off. We identified thermal deformation of the 3D printed PLA stages as the most likely cause. After running the sensor continuously (actively or in standby) thermal expansion was expected, although not as much as encountered deformation. After adapting our algorithmic approach, we tracked the ROI from 2D image features, so the data could still be extracted using the 2D vision for the first 19 patients. This did not affect the time-dependent spectral analysis of heart-rate related color fluctuations. In response to the de-calibration, we decided to perform calibrations regularly every 3 days to avoid this problem. As a result, we could obtain usable 3D data in the second phase.

Furthermore, skin temperature, transcutaneous bilirubin, and the illumination level were documented. For obtaining the transcutaneous bilirubin, the *JM-105* device from Dräger was used. For skin temperature a contactless thermometer (*Beurer FT 90*) was used. The only light sources were natural light from a window to an adjacent room with daylight and artificial light from fluorescent lamps. To provide standardized conditions, the ambient light level was measured *via* a light meter (*PeakTech 5,086 Digital LED LUX Meter*). Data obtained were analyzed after reaching the previous determined number of newborn infants.

### Participants

Eligible for the study were healthy newborn and near-term preterm infants without any cardiac or pulmonary impairment in order to exclude an impact on these pilot measurements. To further avoid potential inaccuracies due to the transition from fetal to neonatal circulation, only infants older than 24 h were admitted to the study. During the time period, the measurements were conducted, all the maternity ward patients were screened for eligibility. Afterward, every legal guardian of the eligible patient was informed about this study and asked for participation. The study was well–accepted, and in only one case, the informed consent was rejected.

To study the potential effects of the different skin temperatures on the accuracy of the measurements, the skin temperature was obtained using a contactless thermometer. Since initially the newborns were placed under a heat lamp, the non-contact thermometer repetitively showed implausible values, so it was decided to proceed without the heat lamp. For the analysis of the temperature these data were excluded.

### Statistical methods

**Figure 5:**
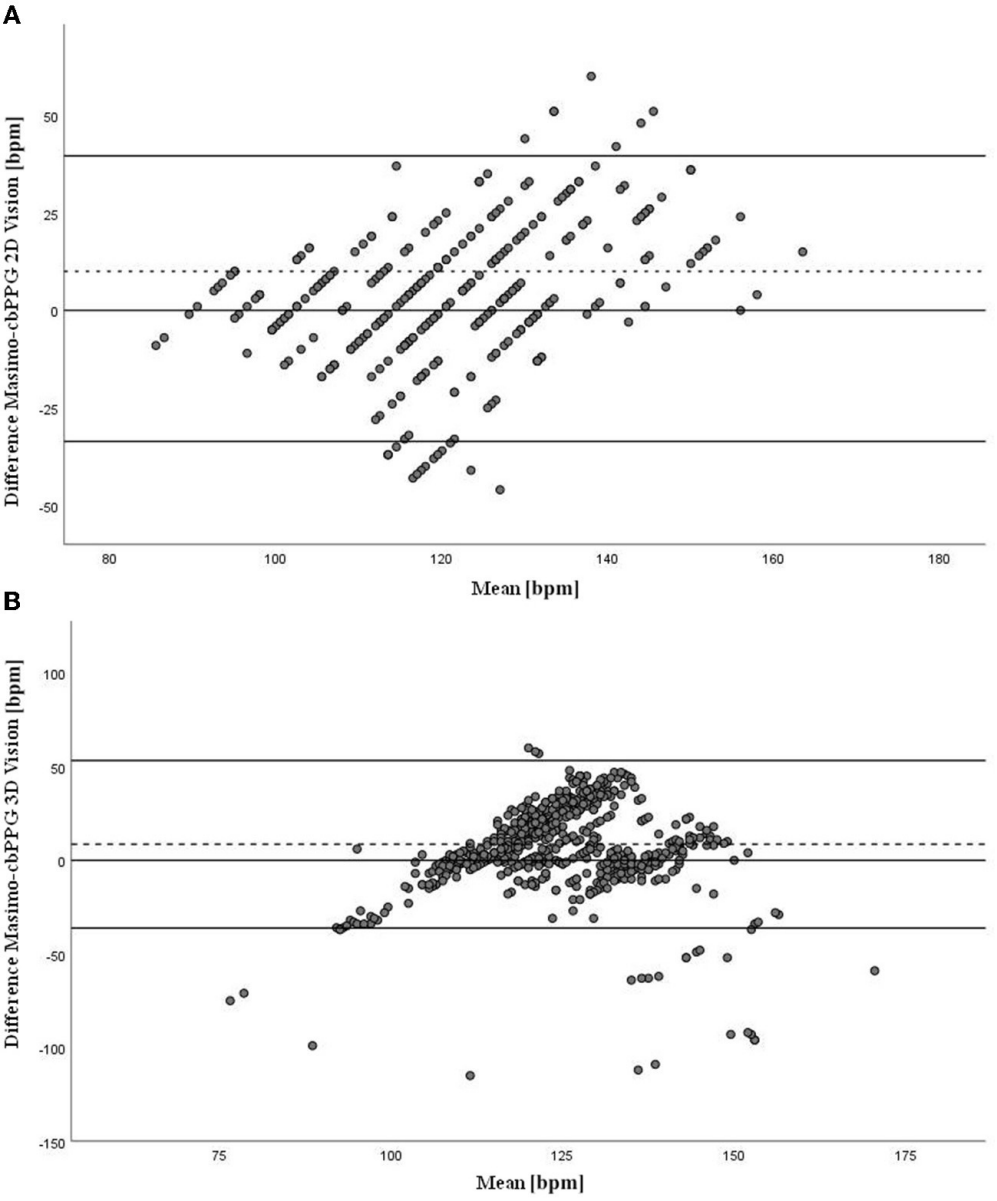
**(A)** Bland-Altman plot shows the level of agreements between the measured HR of Masimo-sensor and the camera-based sensor. The y-axis shows the difference between the two methods (bpm), x-axis shows the mean of the two methods (bpm) (2D vision). **(B)** Bland-Altman plot shows the level of agreements between the measured HR of Masimo-sensor and the camera-based sensor. The y-axis shows the difference between the two methods (bpm), x-axis shows the mean of the two methods (bpm) (3D vision).

Descriptive statistics (mean ± standard deviation for normally distributed data, median and 25th/75th percentile for heart rate, which is not normally distributed, absolute and relative frequencies for gender) were used to summarize the analyzed population. Agreement between measurements of Masimo-sensor and the camera-based sensor was assessed by fitting a linear mixed model with random intercept per patient for the difference of the two systems (Masimo-sensor minus camera-based sensor). By applying this model we account for the multiple measurements per patient and the correlation of these observations. Estimated mean difference with 95% confidence interval is reported to evaluate the agreement of both devices. The mean difference is a frequently used estimate of the bias, i.e., the disagreement of both methods. The 95% confidence interval describes the uncertainty about this estimate, providing a range of possible values regarding the bias. Additionally the level of agreements between the measured HR of Masimo-sensor and the camera-based sensor were assessed using Bland-Altman plots (Figure 5A for 2D vision and Figure 5B for 3D vision). To assess the influence of various parameters on the measurement difference, the linear mixed model was also applied including age, gestational age, weight, skin temperature, transcutaneous bilirubin, illumination and gender as fixed effects and random intercept per patient. The significance level was set at α= 0.05. All statistical analyses were performed using SPSS (IBM Corp. Released 2017. IBM SPSS Statistics for Windows, Version 25.0. Armonk, NY: IBM Corp.).

## Results

By examining our primary objective, we analyzed the feasibility and accuracy of the heart rate measurements of the novel cbPPG-based sensor compared to Masimo pulse oximetry-based monitoring system. A total of 42 infants were studied, mean gestational age was 39.6 ± 1.3 weeks (ranging from 36 to 41 weeks) and average weight was 3167.4 ± 444.8 g (ranging from 2335 g to 4080 g). 24 (57.1%) of the infants were male and 18 (42.9%) female. The light level varied from 290 lx to 365 lx with a mean of 330.4 ± 13.9 lx (see Table 1). A total of 108 measurements were recorded. Nevertheless, some had to be removed partially or entirely from evaluation due to missing intervals. In 6 subjects an extra measurement had to be conducted due to technical problems and completely missing data of pulse oximetry. As mentioned earlier, in the first phase of the study (19 subjects) only the 2D vision was available, whereas, in the second phase (23 subjects) both data using 2D and 3D vision could be obtained. Due to the different methods, we decided to analyze the accuracy of both study groups separately. In the 2D vision group, the mean difference of the measurements of the Masimo device was on average 3.0 bmp (beats per min) higher than those from the camera-based sensor (95% CI −3.7 to 9.7, LOA ± 36.6). According to the statistical analysis, the difference in the results is not significant between the two methods (p = 0.359). In the 3D vision group, the mean difference was 8.6 bpm (95% CI: 2.3 to 14.9, LOA ± 44.7), which is significant (p = 0.010) (see Table 2). We also analyzed the effect of age, sex, gestational age, weight, skin temperature, transcutaneous bilirubin, and illumination on the accuracy of the measurement. None of these effects reached clinical significance, thus accuracy was not affected by these factors (see Table 3).

**Table 1:** Demographic and clinical characteristics of the study population.

**Parameter**	***N***	Mean ±SD ^+^ /Median (25^th^-75th Percentile) ^*^
Gestational age [weeks]	42	39.6 ± 1.3 ^*^
Age [hours]	42	41.1 ± 13.6 ^+^
Weight [g]	42	3167.4 ± 444.8 ^+^
Skin temperature [°C]	38	37.3 ± 0.4 ^+^
Transcutaneous bilirubin [μmol/l]	42	120.5 ± 53.3 ^+^
Illumination [lx]	42	330.4 ± 13.9 ^+^
Heart rate [bpm]	42	127 (113-138) ^*^

*Data marked with + correspond to Mean ± SD. Data marked with*

* * correspond to median (25th-75th Percentile)*.

**Table 2:** Mean difference in heart rate in beats per min comparing Masimo vs. camera-based sensor.

**Parameter**	**Estimate**	**95% confidence interval**	***p* value**
Mean difference Masimo vs. camera-based sensor 2D-Vision [bpm]	3.0	−3.7 to 9.7	0.359
Mean difference Masimo vs. camera-based sensor 3D-Vision [bpm]	8.6	2.3 to 14.9	0.010

**Table 3:** Effects of demographic and clinical characteristics on the accuracy of the heart rate measurements.

**Parameter**	**Estimate**		**95% confidence interval**		***p*** **value**	
**Vision**	**2D**	**3D**	**2D**	**3D**	**2D**	**3D**
Age [hours]	0.2	−0.02	−0.7 to 1.1	−0.8 to 0.8	0.664	0.948
Gestational age [weeks]	−5.2	−4.0	−15.7 to 53	−13.3 to 5.3	0.268	0.370
Weight [g]	0.01	0.009	−0.03 to 0.05	−0.02 to 0.04	0.508	0.461
Skin temperature [°C]	−3.2	4.6	−25.9 to 19.6	−26.4 to 36.6	0.745	0.757
Transcutaneous bilirubin [μmol/l]	−0.05	−0.06	−0.2 to 0.1	−0.4 to 0.2	0.453	0.667
Illumination [lx]	−0.4	−0.03	−1.2 to 0.3	−0.8 to 0.7	0.177	0.926
Male vs. female	−14.6	−10.3	−34.9 to 5.8	−29.0 to 8.4	0.128	0.255

## Discussion

Our pilot study investigated the feasibility and accuracy of an emerging method of contactless heart rate monitoring of newborns using a multimodal camera-based sensor. As reference standard pulse oximetry was used. Our approach is different from the previous studies shown (4, 5, 7, 18–22) as we added 3D vision and compared its accuracy to the 2D method. In the 2D vision group a good agreement between the cbPPG and the pulse oximetry was reached (mean difference 3.0 bpm). This result matches the medical standards (34). However, contrary to our expectations, the 3D vision provided less accurate results (mean difference + 8.6 bpm). The hypothesis that inaccuracy of 3D vision was caused by higher susceptibility to motion artifacts could not be confirmed. Although no commonly significant correlation was found, violent movements are likely connected to individual measurement failures. Regarding the feasibility of the approach, we proved good feasibility with respect to clinical safety, acceptable accuracy, general acceptance, and possible implementation in the clinical routine. In particular, cbPPG presents a non-obtrusive data acquisition tool, capable of being run in parallel to and without disturbing daily clinical practice. This clearly shows promise for future applications.

**Figure 6:**
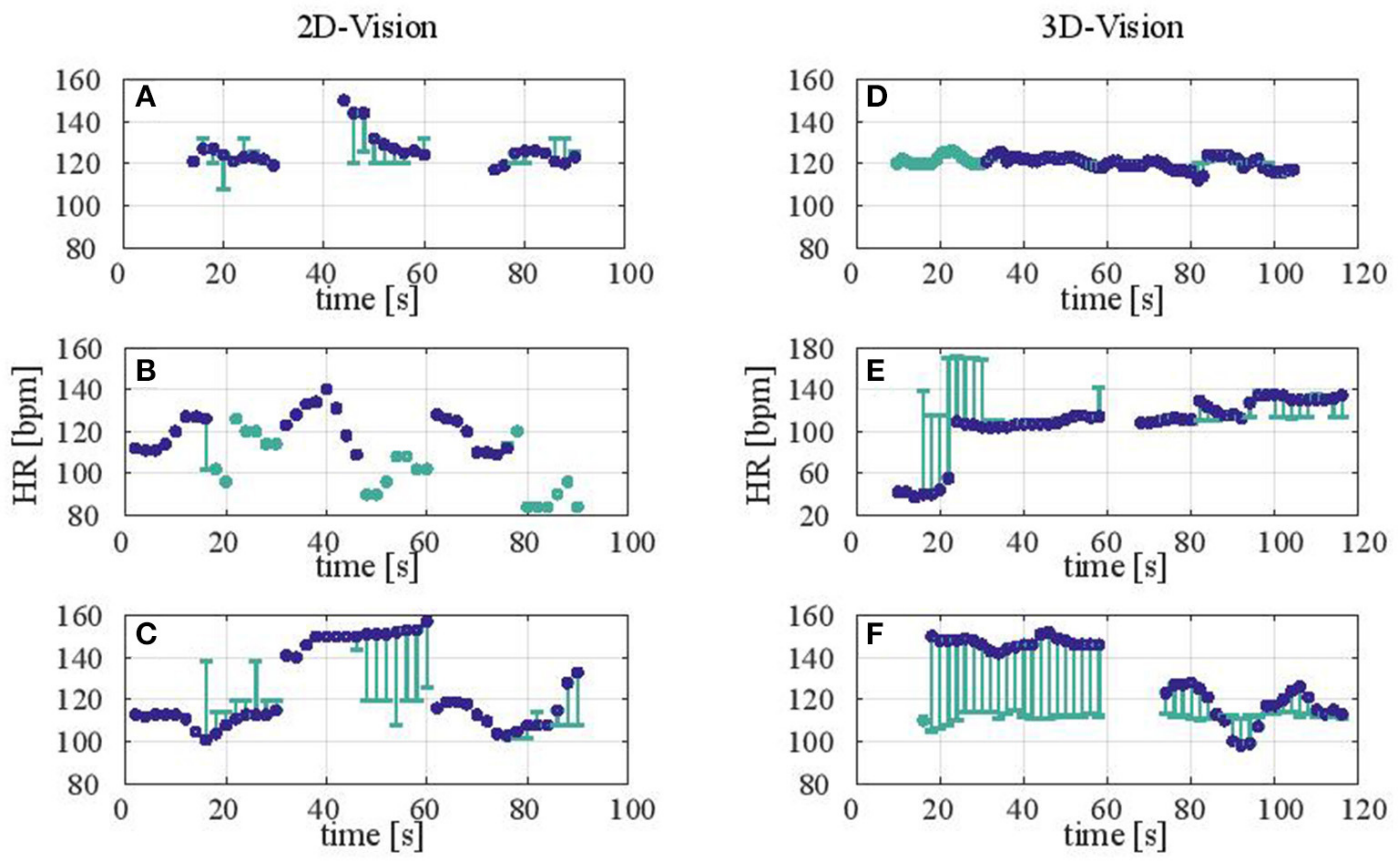
**(A–F)** Representative measurement results comparing 2D and 3D vision (left and right column). Each plot shows the measured or calculated HR in bpm (beats per minute) of a newborn. The data encompasses the concatenated time intervals of all measurements performed on the respective patient. Here, each HR-value corresponds to an average over 2s. Blue circles denote HR values resulting from the reference Masimo pulse oximeter. Teal-colored circles and lines denote HR values resulting from the camera sensor. Whereas the teal circles show data points without a reference (i.e., the Masimo device failed to produce data), error bars show how much the camera-based data deviated from the HR measured by the Masimo device.

Currently, the device lacks the accuracy for clinical decision-making. Nevertheless, comparable measurements were achieved with cases of very good agreement under suitable measurement conditions, as shown by our statistical evaluation. For deeper insight into the data underlying the statistics we present representative plots for six patients out of the 42 in total in Figure 6. While comparing 2D and 3D vision, we can clearly see measurement events where HR values from both devices produce comparable results (Figures 6A,D). However, we can also observe significant deviations from the reference in (Figures 6C,E,F). A closer look at each data set reveals important details about the different properties of each measurement as well as the potential for future studies. First, we see in Figure 6A that Masimo and camera-based results are clustered together, with missing data points in between. This indicates that both devices suffered from the same or similar perturbations (such as movement artifacts). In Figure 6B, we see that the data sets alternate, giving the impression that the camera-based data is a continuation of Masimo results. It is warranted that further reference measurements would be needed to ensure the validity of these intermediate results. It is, nevertheless, an interesting perspective that a contactless measurement could step in where cable-based sensors fail. Similarly, in the center of the plot of Figure 6C we observe a jump in the HR values of the Masimo device, whereas the camera-based results indicate a more continuous distribution of HR values around 120 bpm. The same can be found in Figure 6F, where the camera system measured close to 120 bpm, but Masimo indicates more fluctuation and jumps across all time intervals. Finally, regarding (Figure 6E) we can see that both data sets were perturbed at the beginning of the measurement but later converged to the same HR value. All in all, this shows that both cable-based, and contactless pulse oximetry can show similar measurement behaviors in some cases, while in others they are susceptible to different confounding factors.

Upcoming studies will be conducted alongside monitoring in the NICU, examining preterm infants or compromised newborns for longer periods, validating the results on more patients, and to improve the systems' robustness with respect to patient movement artifacts.

## Limitations

There are some limitations of our study we have to mention. Firstly, as alluded earlier, due to the deformation of the parts build of polyactic acid (PLA), the data using 3D vision could not be obtained in the first phase. This problem was fixed on the second phase by performing regular calibrations every 3 days. Secondly, the HR was derived from pulse oximetry, no ECG was used. ECG is considered to be the gold standard in obtaining the HR and would possibly replace missing intervals in the pulse oximetry. However, the available ECG monitors did not provide the possibility of data storage at the required intervals. Thirdly, considering our study population, our findings currently may be applied only for healthy newborns and near-term preterm infants with normal heart rate. Fourthly, we used standardized illumination, thus lighting artifacts which were described as one of the disruptive factors (23, 24) could be excluded. Crepuscular light, however, could be a limitation in the neonatal intensive care unit (NICU), because increased illumination would interfere with the standards of minimal handling and disrupt the sleeping cycle of the neonates. Fifthly, another potential source of measurement inaccuracy that remains unsolved was swarthiness, as all the newborns examined in the study were Caucasian. Sixthly, the data from the camera-based system were delivered *via post hoc* analysis and thus not constantly available. For the clinical setting a real time imaging system is necessary. Sevently, one of our main concerns was the high rate of missing intervals using the cbPPG-based sensor as well as the pulse oximeter. Our hypothesis is that this is caused by motion artifacts. We intend to investigate the problem further in a following study on the NICU. In order to reduce the frequency motion artifacts and establish conditions for monitoring for longer periods techniques such as swaddling, non-nutritive sucking or oral administration of glucose solution can be applied (35, 36).

## Conclusions

We proved the feasibility and accuracy of a novel method of contactless heart rate monitoring in healthy neonates with normal heart rate using a multimodal camera-based sensor. The use of this technology could have multiple potential benefits; however, extensive further research is warranted.

## Data availability statement

The raw data supporting the conclusions of this article will be made available by the authors, without undue reservation.

## Ethics statement

The studies involving human participants were reviewed and approved by Ethics Committee of the Jena University Hospital. Written informed consent to participate in this study was provided by the participants' legal guardian/next of kin.

## Author contributions

HP accomplished the funding together with MN, JS, and GN. LS, JS, MN, and HP contributed to conception and design of the study. LS conducted the measurements and wrote the first draft of the manuscript. MN performed the statistical analysis. JS supervised the assembly of the camera-based sensor. CZ wrote the software for image acquisition and evaluation. JS, MN, and CZ wrote sections of the manuscript. All authors contributed to manuscript revision, read, and approved the submitted version.

## Funding

We gratefully acknowledge financial support by the German Federal Ministry of Education and Research in the program Zwanzig20–Partnership for Innovation as part of the research alliance 3Dsensation (grant numbers 03ZZ0482A, 03ZZ0482B, and 03ZZ0482C).

## Conflict of interest

The authors declare that the research was conducted in the absence of any commercial or financial relationships that could be construed as a potential conflict of interest.

## Publisher's note

All claims expressed in this article are solely those of the authors and do not necessarily represent those of their affiliated organizations, or those of the publisher, the editors and the reviewers. Any product that may be evaluated in this article, or claim that may be made by its manufacturer, is not guaranteed or endorsed by the publisher.

## Abbreviations

Bpm: beats per min
cb-PPG: camera-based photoplethysmography
CI: confidence interval
EVM: eulerian video magnification
FWHM: full width at half maximum
GOBO: GOes Before Optics
HR: heart rate
LED: light emitting diode
LOA: limit of agreement
NICU: neonatal intensive care unit
NIR: near-infrared
PLA: polyactic acid
PPG: photoplethysmography
ROI: region of interest.
